# Tocilizumab provides a potential therapeutic option for the management of hyperhaemolysis syndrome in sickle cell disease: A case series and brief narrative overview of the literature

**DOI:** 10.1111/tme.70026

**Published:** 2025-10-09

**Authors:** S. Wolf, B. Singh, A. Zaidi, P. Greaves, F. Oyesanya, S. Bennett, B. Kaya, F. Barroso, P. Telfer

**Affiliations:** ^1^ Department of Clinical Haematology Barts Health NHS Trust London UK; ^2^ Red Cell Immunology NHS Blood and Transplant London UK; ^3^ Blizard Institute Queen Mary University of London London UK; ^4^ Department of Clinical Haematology Barking, Havering and Redbridge University Hospitals NHS Trust London UK

**Keywords:** hyperhaemolysis, sickle, tocilizumab

## Abstract

**Background and Objectives:**

Hyperhaemolysis syndrome is a life‐threatening complication of transfusion, potentially triggered by macrophage activation, with limited treatment options. Tocilizumab, an anti‐IL6 monoclonal antibody, has mechanistic rationale for use and has been shown to be effective in a small number of cases. In this paper, we review four cases of hyperhaemolysis treated with tocilizumab in the context of the existing literature.

**Materials and Methods:**

Cases of use of tocilizumab in hyperhaemolysis were identified from two large specialist haemoglobinopathy centres between the period January 2021 and March 2025. Clinical and laboratory data were collected.

**Results:**

Four cases of hyperhaemolysis treated with IVIG, steroids and tocilizumab were reported. In all cases, haemolysis responded rapidly to tocilizumab therapy. Two patients subsequently received RBC transfusions without haemolysis; two patients died from causes unrelated to haemolysis.

**Conclusions:**

This case series supports the use of tocilizumab as a therapeutic option for rapid resolution of haemolysis. It is generally widely available and should be considered a suitable and cost‐effective alternative to currently available options.

## 1 BACKGROUND

Hyperhaemolysis syndrome (HHS) is a rare but life‐threatening complication of transfusion with limited treatment options. It is characterised by rapid haemolysis of both donor and recipient red cells, and most cases are associated with sickle cell disease. While the pathophysiology is unknown, it is believed to represent either an antibody‐mediated complement activation pathway or an activation of macrophages resulting in both destruction of red cells and reticulocytes.^1^

Predisposing factors for HHS include: female sex, non‐B blood group, prior alloimmunisation and immunological activation through infection, pregnancy, or acute chest syndrome^2^, ^3^ and recent evidence has identified potential genetic variants that may predispose to the condition.^4^ Patients typically present 6 or 7 days after receiving red cell transfusions, with symptoms of pain, fever and dark urine and a drop in haemoglobin to a nadir around 40 g/L at 10 days.^3^ Laboratory findings include reticulocytopenia and elevated bilirubin, LDH and ferritin compared to the steady state. Around half of all patients have a negative DAT, and new alloantibodies are detected in around 40% of cases.^3^ These criteria have been incorporated into a provisional set of diagnostic criteria,^5^ which can help aid in the standardisation of diagnosis.

Treatment for HHS has varied, but international guidelines typically recommend steroids and IVIG for first‐line therapy. For refractory cases, rituximab and eculizumab have been used; however, the evidence supporting these therapies is limited, with only six cases of eculizumab use presented as evidence to support commissioning policy of the UK National Health Service. In the case of rituximab, although there is greater experience reported in the literature, clinical responses can be delayed^6^ and there are questions raised about the efficacy of rituximab prophylaxis in the absence of antibody‐mediated transfusion reaction. Pre‐transfusion eculizumab has also failed to prevent HHS when used as prophylaxis in previously affected patients.^7^ Since the UK haemovigilance scheme Serious Hazards of Transfusion (SHOT) began collecting data on HHS in 2020, there has been only one reported case of eculizumab use and no cases reporting the use of rituximab in the management of hyperhaemolysis.^8^

In view of the potential macrophage activation driving HHS, there may be a role for anti‐IL6 therapy. Several case reports have been published demonstrating the effectiveness of the anti‐IL6 monoclonal antibody, tocilizumab. These reports suggest good responses, characterised by a rapid fall in ferritin and bilirubin, with a corresponding rise in reticulocytes and haemoglobin. There is some suggestion that tocilizumab may result in a shorter time to hospital discharge as compared to eculizumab, and no significant side effects or deaths have been reported with its use.^9^, ^10^, ^11^, ^12^, ^13^, ^14^, ^15^, ^16^, ^17^, ^18^ However, to date, there have been no head‐to‐head studies or commercial trials in this area. In this paper, we outline our recent experiences of using tocilizumab in HHS in sickle cell patients, adding to the growing body of evidence supporting its use.

## 2 METHODS

Cases of hyperhaemolysis that had not previously been reported in the literature were collected from two specialist haemoglobinopathy centres known to have treated patients with tocilizumab, from pharmacy records of tocilizumab administration given to Clinical Haematology patients from January 2021 to March 2025, and confirmed by local clinical Haematology teams. Cases were included where tocilizumab was administered to prevent or mitigate a transfusion reaction and excluded where tocilizumab was given for another reason such as Covid‐19 infection. Medical records were evaluated to collect clinical, laboratory and treatment data on these patients.

## 3 RESULTS

Four new cases of tocilizumab use in HHS were identified. Demographic data is shown in Table 1. All patients had the genotype HbSS. Three patients had pre‐existing allo‐antibodies (Table 1), and of these three, all had a history of delayed haemolytic transfusion reactions (DHTR), although all had been transfused subsequently without reaction. In the case of three patients with a history of DHTR, two were transfused as emergencies for acute chest syndrome (Cases 2 and 3) and one was transfused electively for symptomatic cerebral vasculopathy (Case 4). The decision to transfuse this patient electively despite multiple antibodies was complex and discussed by a multi‐disciplinary team including neurologists, haematologists and radiologists. The median units transfused in each case were 2 (range 1–3). All units were ABO compatible, matched for Rh and K, sickle negative and negative for any clinically significant pre‐existing antibodies, as per the BSH guidelines for transfusion in sickle cell disease.^19^ In the case of a patients with antibodies to Leb (Case 3), antigen‐negative units were not selected, in line with NHS Blood and Transplant recommendation that in the case of anti‐Lea antibodies, red cells can be issued that are cross‐match compatible at 37°.^20^ Cross‐matching was carried out by indirect antiglobulin test (IAT) (column agglutination technique) except in Case 1 where units were issued electronically due to lack of previous antibodies. Where cross‐match was performed, all units were cross‐match compatible.

**TABLE 1:** Details of patients and previous transfusion history.

Case	Age	Sex	Genotype	Blood group	Previous allo‐antibodies	Previous transfusion reaction	Indication	No. of units	Pre‐transfusion Hb (g/L) (NR 120–160 (f), 130–170 (m))	Post‐transfusion Hb (g/L)
1	26	F	HbSS	A RhD−	None	None	Pre‐operative optimisation	2	73	Not done
2	20	F	HbSS	A RhD+	Y (anti‐Fya, anti‐S, anti‐Jkb)	DHTR 9 years prior, transfused regularly since with no reaction	Acute chest syndrome	3	62	79
3	26	M	HbSS	B RhD+	Y (anti‐S, anti Jka, anti Leb)	DHTR 9 years prior; transfused twice since with no reaction	Acute chest syndrome	1	65	63
										
4	34	M	HbSS	O RhD+	Y (anti‐S, anti‐Fyb, anti‐Jkb)	DHTR 8 years prior; transfused subsequently without reaction	Symptomatic cerebral vasculopathy	2	91	Not done

Abbreviations: DHTR, delayed haemolytic transfusion reaction; F, female; Hb, haemoglobin; M, male.

After initial presentation, all patients were discharged and subsequently re‐presented to the hospital. Clinical and laboratory data from the time of presentation with HHS are given in Table 2. All patients presented with severe pain, with dark urine observed typically a few days later. Fever was observed on presentation in three cases. Blood results included a positive DAT in all four cases, details of which are in Table 2. No new allo‐antibodies were identified except in Case 2, where a new allo anti‐C was detected 4 weeks later. Subsequent follow‐up for a minimum of 18 months in all surviving cases has not detected any new antibody formation.

**TABLE 2:** Details of clinical presentation post‐transfusion.

Case	Days post‐first RBC	Presentation	Hb (g/L) (NR 120–150 (f), 130–170 (m))	DAT	Reticulocytes (× 10^9^/L) (NR 50–100)	Bilirubin (μmol/L) (NR 0–21)	Hb nadir (g/L)	Ferritin, peak (mcg/L) (NR 13–150)	Ferritin, pre‐tocilizumab (mcg/L)	Tocilizumab given day (post‐RBC)	Ferritin 48 h post‐tocilizumab (mcg/L)
1	19	Pain, fever, dark urine	53	IgG 1+	217	118	35	17 034	17 034	20	3542
2	6	Knee and back pain, fever	66	IgG 1+	43	42	25	>100 000	14 300	14	11 038
3	7	Chest, knee pain	49	IgG 1+	130	46	33	1637	1637	7	Not done
											
4	4	Leg and back pain, fever	94	IgG 3+ C3d 1+	231	215	50	25 566	25 566	6	6798

*Note*: The date of initial RBC transfusion is considered D0.

Abbreviations: DAT, direct antiglobulin test; Hb, haemoglobin.

**FIGURE 1:**
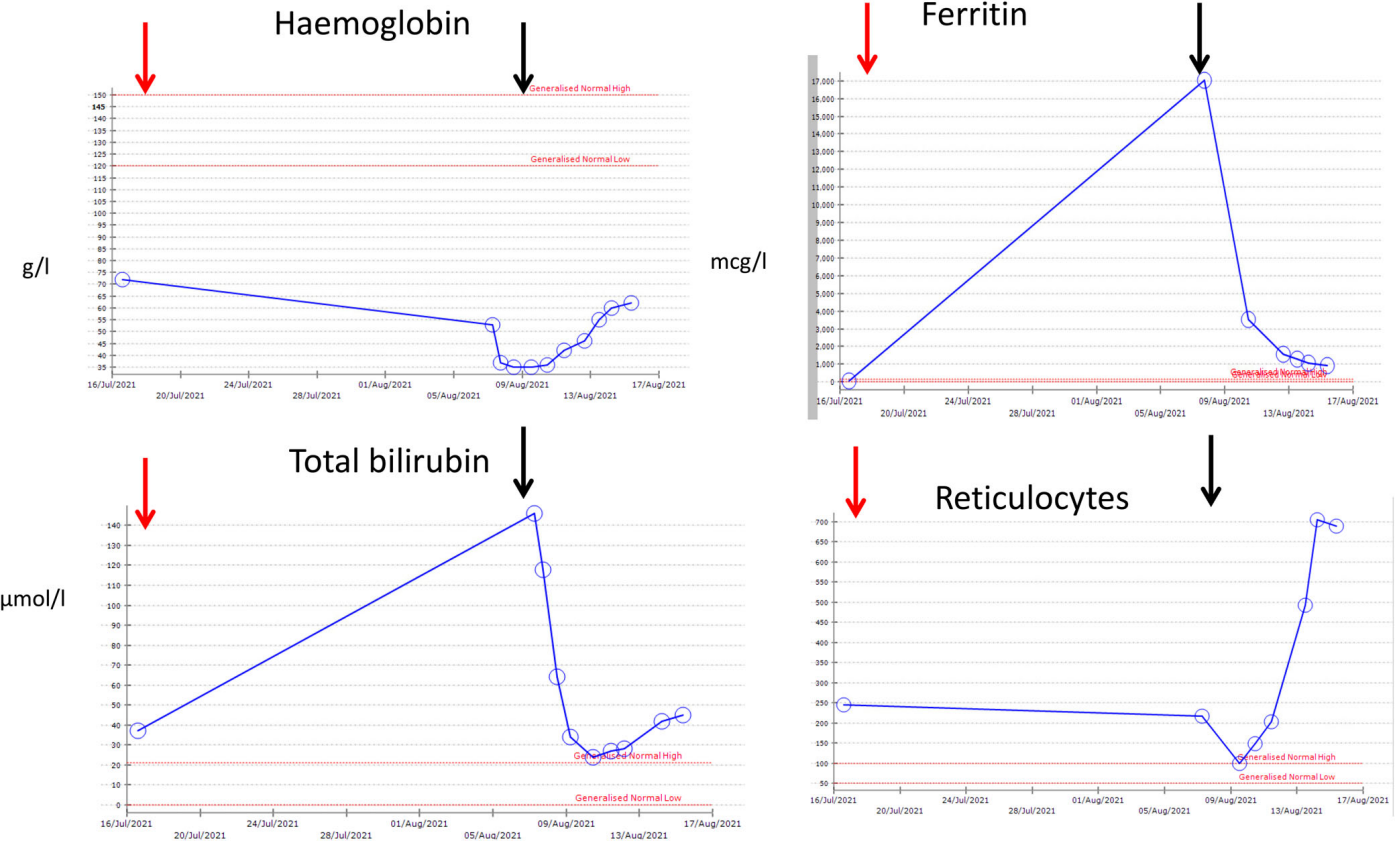
Response of haemoglobin, ferritin, bilirubin and reticulocytes to transfused red cells (red arrow) and tocilizumab (black arrow). Patient was discharged from hospital the day after the last data points are shown.

All patients were treated initially with IVIG and steroids, as per local policy, and were subsequently treated with tocilizumab, all at a dose of 8 mg/kg. Case 4 received a second course of tocilizumab. Cases 1 and 2 did not require any further red cell transfusion. The response of Case 1 to tocilizumab is demonstrated in Figure 1, with a rapid fall in ferritin and bilirubin, and a rise in reticulocytes and haemoglobin in the subsequent few days. Falls in ferritin, where measured, are observed in all cases within 48 h following tocilizumab (Table 2). Case 3 deteriorated with worsening urine output and was transfused five units of red cells with IVIG, on D8 after his initial transfusion and the day after he re‐presented to hospital and was given tocilizumab. He was being transferred to intensive care when he had a cardiac arrest during transfer. He was resuscitated but developed renal failure and died the following day. A subsequent post‐mortem showed recent ischaemic damage to the heart and evidence suggestive of pulmonary hypertension. Case 4 developed fluctuating consciousness with agitation and developed an increased oxygen requirement. There was no new intracranial pathology, and he was transfused one unit of red cells after the second dose of tocilizumab. Despite his markers of haemolysis stabilising, he continued to desaturate, requiring intubation, developed multi‐organ failure, and died on day 8 of admission. The presumed cause of death was pulmonary embolism, but post‐mortem was not carried out due to family wishes. Cases 1 and 2 all survived to discharge after a total of 11 and 43 hospital days from readmission. No adverse events relating to tocilizumab were noted.

## 4 CONCLUSION

The addition of these four cases takes the total of reported cases of hyperhaemolysis treated with tocilizumab to 15. This is more than the reported cases of eculizumab (six case reports) on which the NHS commissioning decision was made. In some of the above‐mentioned cases, tocilizumab was given following failure of eculizumab, but in several cases, tocilizumab was given as immunomodulatory therapy after IVIG and steroids.^9^, ^10^, ^11^, ^12^, ^14^, ^15^, ^16^, ^17^ Duration of therapy ranged from one to 4 days; however, in our experience, we have found that a single dose at 8 mg/kg is sufficient.

No adverse events were observed in the previously reported cases, apart from one seizure episode which was likely related to methaemoglobinaemia related to the use of HDOC‐21, a polymerised bovine haemoglobin.^10^ The consensus of the multi‐disciplinary team was that the death during the transfer of Case 3 is unlikely to be related to its use. We acknowledge that in Case 4 the inflammatory cascade triggered by transfusion may have contributed to the multi‐organ failure and subsequent death, but there is no indication that tocilizumab further contributed to this, especially as haemolysis settled very rapidly following the tocilizumab infusion (Table 2). It is notable that in three of the four cases, there was a history of transfusion reaction and/or allo‐antibodies, but in all cases, the transfusion was deemed necessary by a multi‐disciplinary team and serves as a tragic reminder that transfusion is still not without risk, even when mitigated as far as is possible. In a brief overview of cases where eculizumab has been used to treat hyperhaemolysis in adults, we note 2 deaths in a total of 12 cases (17%).^7^, ^21^, ^22^, ^23^, ^24^, ^25^, ^26^, ^27^, ^28^, ^29^ This is comparable to a total of 2 deaths (reported here) in a total of 15 cases of tocilizumab use in adults (13%).^9^, ^10^, ^11^, ^12^, ^13^, ^14^, ^15^, ^16^, ^17^ There is insufficient data available to comment on the safety of either drug in children for hyperhemolysis, although both are in use for alternative indications.

The most reported side effect of tocilizumab is upper respiratory tract infection, and serious infections, while they do occur, are rare. We did not observe any in this cohort. Eculizumab carries an increased risk of meningococcal infection, requiring pre‐emptive vaccination as far as possible, while this is not the case for tocilizumab. In our institutional experience, tocilizumab is generally easier to acquire than eculizumab and has a significantly lower cost in the UK,^30^ although we acknowledge that this may vary by setting. There is also some suggestion that hospital days are shorter than with eculizumab.^3^

We suggest that the use of tocilizumab *could be* considered as an alternative to eculizumab in uncomplicated HHS in sickle cell disease, where there is evidence of an inflammatory response, indicated by fever or macrophage activation, as previously described,^12^ and we encourage the dissemination and publication of similar experiences, and ultimately a larger‐scale randomised controlled trial in order to compare therapies. This *may ultimately lead* to a change in commissioning policy and a wider range of options available for management of hyperhaemolysis.

## AUTHOR CONTRIBUTIONS

SW, PT, and FB conceived the idea of the paper; SW, BS, AZ, PG, FO, BK, and FB contributed data; SW wrote the paper, and PT approved the final draft. All authors reviewed the manuscript and provided feedback.

## CONFLICT OF INTEREST STATEMENT

The authors have no competing interests.

## PATIENT CONSENT STATEMENT

Informal verbal consent to treat patients as per institutional guidelines was provided.

## Data Availability

Further data is available on request from the authors.
